# Graphene Modified TiO_2_ Composite Photocatalysts: Mechanism, Progress and Perspective

**DOI:** 10.3390/nano8020105

**Published:** 2018-02-12

**Authors:** Bo Tang, Haiqun Chen, Haoping Peng, Zhengwei Wang, Weiqiu Huang

**Affiliations:** School of Petroleum Engineering, Changzhou University, Changzhou 213016, China; tangbo@cczu.edu.cn (B.T.); penghp@cczu.edu.cn (H.P.); wangzhw@cczu.edu.cn (Z.W.); hwq213@cczu.edu.cn (W.H.)

**Keywords:** photocatalyst, graphene, TiO_2_, electron transport

## Abstract

Graphene modified TiO_2_ composite photocatalysts have drawn increasing attention because of their high performance. Some significant advancements have been achieved with the continuous research, such as the corresponding photocatalytic mechanism that has been revealed. Specific influencing factors have been discovered and potential optimizing methods are proposed. The latest developments in graphene assisted TiO_2_ composite photocatalysts are abstracted and discussed. Based on the primary reasons behind the observed phenomena of these composite photocatalysts, probable development directions and further optimizing strategies are presented. Moreover, several novel detective technologies—beyond the decomposition test—which can be used to judge the photocatalytic performances of the resulting photocatalysts are listed and analyzed. Although some objectives have been achieved, new challenges still exist and hinder the widespread application of graphene-TiO_2_ composite photocatalysts, which deserves further study.

## 1. Introduction

As one of the low-cost technologies in the field of environmental protection, tremendous developments in both the theories and experiments of photocatalysis have been achieved because of the worsening pollution problem [1,2,3,4,5,6,7,8,9,10]. Among the different types of semiconductors (TiO_2_, ZnO, CdS, WO_3_ et al.), TiO_2_ draws additional attention because of its low-toxic, high activity and excellent chemical stability [11,12]. However, two bottlenecks, including its lack of response to visible light and the high recombination rate of electron-hole pairs, hinder its widespread application [13]. The primary causes are the wide band-gap of TiO_2_ (~3.2 eV, the onset wavelength is ~390 nm) and the short mean free path of electrons in this material [14,15,16]. Scientists and engineers have made efforts to conquer these two shortages and all the adopted approaches can be classified into two types: internal doping and surface sensitization [17,18,19,20,21,22,23,24,25,26,27,28,29,30]. An impurity level will be introduced to the band-gap of TiO_2_ after adding metal or non-metal ions. Zheng et al. reported that the band-gap of N doped rutile TiO_2_ decreases into 1.553 eV according to the first-principles calculation [25]. Zhou’s group found that the impurity level of N, La co-doped TiO_2_ is ~0.3 eV lower than the conduction band of the pure TiO_2_ (the absorption band exhibits a red shift to 436.4 nm) [26]. 

Although the formation of a new energy level endows visible light activity to TiO_2_, the additional impurity ions simultaneously act as recombination centers for photo-generated electron-hole pairs [17,18,19]. Therefore, surface sensitization is considered a preferred strategy for the modification of TiO_2_ with fewer negative effects [14,15,27,28,29,30]. Selecting a proper sensitizer is key and some pre-conditions should be satisfied. First, an elaborate selection (or design) is needed for the electronic structure of a sensitizer. Besides the band-gap of the adopted material (which can be excited by the visible light) should be narrower than that of TiO_2_, the conduction band of the sensitizer must be more positive than that of TiO_2_ (or the valence band of the sensitizer is more negative than that of TiO_2_). Moreover, not only can the combination of the TiO_2_ and sensitizer be realized by convenient methods but also the loading amount of the sensitizer should be controllable. Therefore, appropriate dangling bond and morphology of a sensitizer need to be considered. Camarillo et al. prepared (Pt, Cu)-TiO_2_ composite photocatalysts to convert CO_2_ to hydrocarbons (as the fuel) with resulting high performance [31,32,33,34]. Chowdhury et al. reported an eosin Y dye sensitized TiO_2_ photocatalyst with high visible light activity [35]. Kukovecz’s group adopted PbSe quantum dot as the sensitizer to modify TiO_2_ nanowires and the resulting composite displays high performance under visible light illumination [36]. Recently, various allotropes of carbon materials, including active carbon, carbon nanotubes (CNTs) and graphene, have been employed to combine with TiO_2_, opening a door to a research frontier for this traditional semiconductor material [37,38,39,40,41,42,43,44]. Yu et al. prepared the C_60_ modified TiO_2_ and the photocatalytic oxidation rate of gas-phase acetone is 3.3 times higher than that achieved when adopting the P25 [38]. Woan et al. reported that the CNTs (including metallic, semiconducting and defect-rich samples) assisted TiO_2_ and the chemical bond between the CNTs and TiO_2_ was found to be a key factor in the resulting high photocatalytic performance [45]. Vajda et al. further appraised the sensitization effects of the single-wall CNTs and multi-wall CNTs with different mass fractions [46].

**Figure 1:**
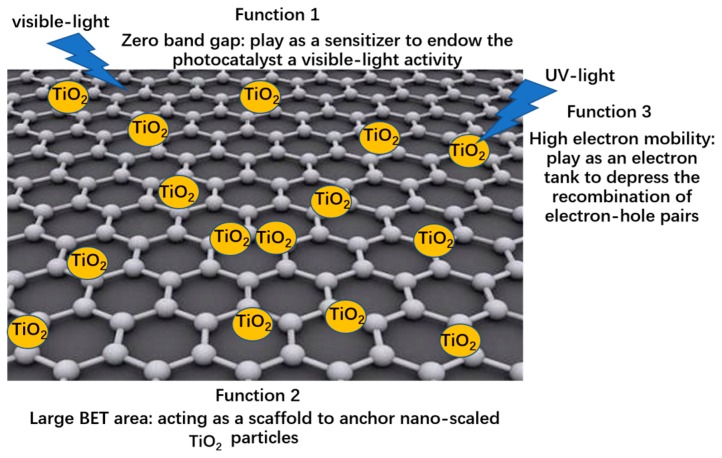
Three major functions of graphene in the resulting composite photocatalysts.

Graphene has become a ‘star’ material since its isolation by Geim and Novoselov for the first time in 2004 and since then, the preparation and applications of this strict two-dimensional material have quickly attracted intensive attention [47,48,49,50,51]. The high electron mobility, a large Brunauer-Emmett-Teller (BET) specific surface area, excellent thermal conductivity and outstanding mechanical strength make graphene a versatile material [52,53,54,55,56]. Naturally, graphene is deemed a promising modifier for photocatalysts, based on its unique properties—Figure 1 displays the major functions of graphene in the resulting composite photocatalysts. First of all, the zero band-gap (semi-metal) of graphene provides the pre-condition for a perfect sensitizer (photo-induced electrons can be excited on the Fermi level of graphene by visible light and infrared irradiation) and its high electron mobility—which results from delocalized conjugated π electron—is beneficial to the resulting photocatalytic performance [47,57,58]. Zhang et al. reported that the π*-d* electron coupling realizes the fast transport of the photo-induced electron between graphene and TiO_2_, which efficiently suppresses the recombination of the photo-generated electron-hole pairs in TiO_2_ [59]. 

Secondly, a large BET area of graphene not only offers a favorable scaffold with which to anchor TiO_2_ nanoparticles but also enhances its adsorption ability for various pollutants [60]. Xu et al., Kamat et al. and our group reported that the P25, titanate nanotubes (TNTs) and silver nanoparticles can symmetrically distribute on the graphene surface [15,60,61]. Thirdly, the high electron mobility of graphene endows it with a great electron tank to promote the separation of electron-hole pairs. Lastly, the efficient combination of graphene and TiO_2_ can be achieved by way of a facile hydrothermal method [62,63,64,65,66]. Other methods including supercritical reaction, chemical vapor deposition (CVD) and self-assembly growth etc. are suggested for the preparation of graphene-TiO_2_ nanocomposites [31,32,33,34,67,68,69,70,71,72]. Camarillo and Tostón et al. found that a remarkable enhancement of the CH_4_ production rate can be achieved when supercritical fluid technology is adopted [32,33]. Kim et al. developed a self-assembly technology to prepare the graphene-TiO_2_ composite with a core-shell structure and the improved photocatalytic activity results from the enhanced charge separation ability [67]. 

**Figure 2:**
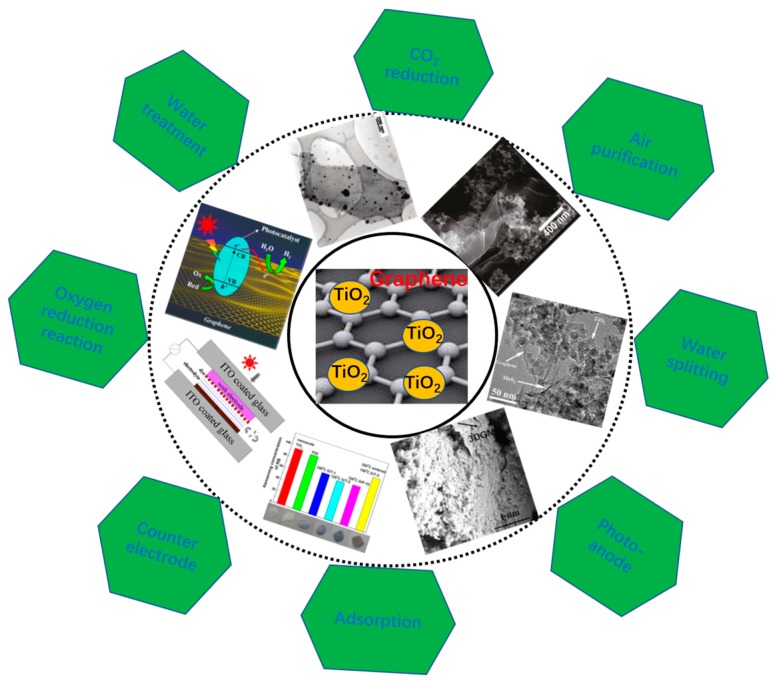
Overview of different research areas of graphene-TiO_2_ composites. Reproduced with permission from [15]. Copyright Elsevier, 2011. Reproduced with permission from [75]. Copyright ACS, 2013. Reproduced with permission from [76]. Copyright ACS, 2013.

Shao’s group synthesized graphene directly over an atomically flat TiO_2_ surface using the CVD method to avoid the presence of contamination at their interface [68]. Chen et al. prepared a graphene-TiO_2_ composite by using TiCl_3_ and graphene oxide as the raw materials and the resulting photocatalyst demonstrates high performance because of the formation of p-n heterojunction between graphene and TiO_2_ [70]. Although these approaches possess respective advantages, the hydrothermal method is the most popular way to fabricate graphene-based composites because of the high yield and low cost. The application area of graphene-TiO_2_ nanocomposites is not limited in photocatalysis and these materials are widely utilized in solar cells and supercapacitors, which have been discussed in some other reviews (Figure 2) [4,73,74,75,76,77,78,79,80]. Moreover, the high photocatalytic performances of the resulting graphene-TiO_2_ composite photocatalysts under both the UV- and visible light irradiation have been reported and the corresponding theories and mechanisms have been discussed [14,15,81,82]. However, some obvious deficiencies have gradually been exposed with continuous research. Firstly, the actual BET area of the widely adopted graphene (reduced graphene oxide, RGO) is ~50 m^2^·g^−1^, which is only 2% of its theoretical value (~2600 m^2^·g^−1^) [83,84]. The small BET area limits the adsorption amount for pollutants, which goes against a high decomposition rate. In addition, the high defect density of the RGO decreases the mean free path of electrons (exerts a negative effect on the lifetime of photo-induced electrons), confining the resulting photocatalytic activity [63,65,66,83]. At last, the uniformity of the RGO (thickness and size) is difficult to ensure, which depresses the sufficient contact between the graphene basal plane and TiO_2_ particles (degraded the photocatalytic performance) [64,84]. Therefore, aiming at how to enhance their chemisorption ability, depress the recombination of the photo-generated electron-hole pairs and promote the electron transport at the interface of graphene and TiO_2_, some optimization methods have been put forward to boost the photocatalytic performances of graphene-TiO_2_ composite photocatalysts [55,85,86]. 

In this study, the latest progress on the graphene-TiO_2_ composite photocatalyst is reviewed and the probable development directions and tendencies are predicted. In Section 2, recently reported photocatalytic performances and the corresponding photocatalytic mechanisms of the graphene-TiO_2_ composite photocatalyst are abstracted and discussed. In particular, the photocatalytic mechanisms under visible light illumination are emphatically analyzed, including the electron transport path and probability. Various optimization approaches, which are employed to enhance the photocatalytic performance of the graphene-TiO_2_ composite photocatalysts, are described and discussed in Section 3 and the core reasons behind the obtained phenomena are revealed. Specially, the three-dimensional graphene networks (3DGNs) assisted TiO_2_ is put emphasis on discussing. In Section 4, a prospective is provided. The major discussion is organized around three aspects: the improvement of chemisorption ability of the graphene-TiO_2_ composite photocatalyst, the prolongation of the photo-induced electron lifetime in TiO_2_ and the enhancement of the electron transport at the interface between graphene and TiO_2_.

## 2. Overview of Graphene-TiO_2_ Composite Photocatalyst

### 2.1. Photocatalytic Performances of Graphene-TiO_2_ Photocatalyst

Since graphene was found to be a promising carrier for nanoparticles, the two-dimensional material modified TiO_2_ composite photocatalysts have become a hot issue. Zhang et al. adopted a one-step hydrothermal method to prepare chemically bonded RGO-TiO_2_ composite photocatalyst and the resulting decomposition rate constant of methylene blue (MB) significantly enhances [84]. Because of both the reduction process of the RGO and the combination process of the RGO and the TiO_2_ nanoparticles that can be achieved during the hydrothermal reaction, this technology is widely used to fabricate the graphene-TiO_2_ composite photocatalysts [84,87,88]. Moreover, some other approaches have also been adopted to fabricate the graphene-TiO_2_ photocatalysts in recent years. Václav et al. adopted thermal hydrolysis of the RGO nanosheets and titania–peroxo complex to fabricate the RGO-TiO_2_ composites and the resulting sample shows a high photocatalytic performance [89]. Williams et al. provide a facile method to prepare the RGO-TiO_2_ composites and the commonly employed hydrothermal process is replaced by a UV-light irradiation step to achieve the reduction and combination of the RGO and TiO_2_, simultaneously [90]. Miyauchi’s group employed the spin-coating technology to fabricate the gaphene-TiO_2_ thin film on a glass substrate and the composite film displays superhydrophilicity and a high photocatalytic activity [91]. In order to further improve the homogeneous coating of TiO_2_ on the graphene surface, benzyl alcohol was adopted as the linking agent by Xu’s group [92]. The resulting composite photocatalyst possesses an ultra-large 2D sheet-like morphology and displays a high performance for the selective reduction of aromatic nitro compounds to amines in water under ambient conditions.

To further improve the photocatalytic performance of the RGO-TiO_2_ photocatalysts, various optimized designs have been carried out. Exposed crystal plane of the raw material is found imposing a remarkable influence on the resulting photocatalytic performance. Jiang et al. Wang et al. and Gao et al. reported that the photocatalytic performance is enhanced when the exposed facet of TiO_2_ is {001} [93,94,95]. Moreover, doping is a useful method to improve the resulting photocatalytic performance. Yang et al. fabricated surface fluorinated TiO_2_-RGO composites by a one-step hydrothermal process and the resulting photocatalytic performance enhances significantly [96]. Pham et al. adopted Cu-doped TiO_2_ to hybridize with the RGO and the decomposition rate of MB significantly improves compared with that of using a non-doped sample [97]. Safarpour et al. found that a polyvinylidene fluoride ultrafiltration membrane modified RGO-TiO_2_ photocatalyst shows an enhanced hydrophilicity and antifouling properties [98]. Similarly, doping in graphene is also beneficial to the resulting high performances. Liu et al. synthesized N-doped TiO_2_ and N-doped graphene hetero-structure by the hydrothermal method to enhance the resulting visible light activity [99]. The B and N co-doped RGO sample was adopted to combine with TiO_2_ by Jaiswal et al. and the resulting photocatalytic performance is further enhanced [100,101,102]. Besides doping, optimizing morphologies of the resulting photocatalysts also exerts a significant influence on their photocatalytic performances. Ao et al. reported a flower-liked composite photocatalyst based on the RGO and TiO_2_, the novel morphology brings about an enhanced photocatalytic activity [103]. Our group prepared the RGO-TNTs photocatalysts by adjusting hydrothermal reaction conditions (sodium hydroxide was added into the solution to promote the formation of tubular structure). BET area of the resulting photocatalyst is ~6 times higher than that of the traditional graphene-TiO_2_ nanoparticles sample, which is in favor of the better adsorption ability [15]. Perera et al. prepared the RGO-TiO_2_ nanotube composites and obtained a high photocatalytic performance [104]. Li et al. and Kim et al. further fabricated the RGO-TiO_2_ nanofibers composites to enhance the visible light activity [105,106]. Qiu et al. proposed that mesoporous structured TiO_2_ is beneficial to the resulting photocatalytic performance [107]. Further, the core-shell constructed graphene-TiO_2_ composites were prepared to enhance their photocatalytic performance by Haldorai et al. [108]. Moreover, the graphene quantum dots were found a proper choice to obtain the high photocatalytic activity because of the enhanced separation efficiency of the electron-hole pairs in TiO_2_ [109]. Considering the nano-scaled RGO sheets are difficult to form a continuous electron transport network, the 3D graphene has been prepared and applied. Ding et al. and Zhang et al. reported the 3DRGO (RGO aerogel) modified TiO_2_ and the resulting performance is significantly enhanced compared with that of the 2DRGO added samples [110,111]. Furthermore, perfluorophenyl azide is used as a medium to link the 3DRGO aerogel and TiO_2_ nanoparticles, which depresses the agglomeration of TiO_2_ nanoparticles and enhances the resulting photocatalytic performance [112]. The naturally continuous structure and low defect density endow the 3DGNs (prepared by the CVD method) a potential sensitizer. Our group adopted the 3DGNs to hybridize TiO_2_ by a hydrothermal method and both the photocatalytic performances under UV- and visible light illumination enhances significantly [55]. 

### 2.2. Photocatalytic Mechanisms of the Graphene-TiO_2_ Photocatalyst

#### 2.2.1. Under UV-Light Irradiation

**Figure 3:**
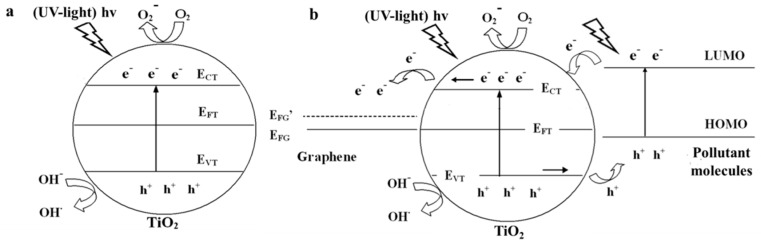
Photocatalytic mechanisms of (**a**) pure TiO_2_ and the (**b**) graphene-TiO_2_ composite under UV-light irradiation. Reproduced with permission from [14]. Copyright Elsevier, 2013.

Photocatalytic mechanism of the pure TiO_2_ has been intensively studied. Under UV-light irradiation (wavelength < 390 nm), photo-induced electrons and holes are excited on the conduction band and valence band of TiO_2_, respectively [113,114]. Then, electrons transfer to surface of TiO_2_ and react with oxygen dissolved in aqueous solution to produce superoxide anion ($O_{2}^{-}$), while holes react with hydroxyl to yield hydroxyl free radical (${\mathrm{OH}}^{\cdot }$). These resulting strong oxidizing radicals play as active substances to decompose pollutant molecules into CO_2_ and H_2_O (Figure 3a). The outputs of $O_{2}^{-}$ and ${\mathrm{OH}}^{\cdot }$ are the most important factors to determine the resulting photocatalytic performances of photocatalysts, which are controlled by the recombination rate of the electron-hole pairs. Therefore, adopting a proper electron tank to promote the separation of the election-hole pairs in TiO_2_ is a powerful method to improve the resulting photocatalytic performance. Graphene should be a competent material for this purpose compared with other carbon allotropes because of its highest electron mobility in theory. Moreover, photo-generated electrons in the conduction band of TiO_2_ would spontaneously transport into graphene due to the more positive Fermi level of the former (work function is 4.6 eV for graphene and 4.2 eV for TiO_2_) [14,115,116]. It is worth to note that the band bending at the interface of graphene and TiO_2_ can be ignored due to their size (nano-scaled average size), which will be further discussed in Section 2.2.2. Therefore, graphene is actually an excellent tank to storage the photo-induced electrons transported from TiO_2_ and the corresponding photocatalytic mechanism is displayed in Figure 3b. 

#### 2.2.2. Visible light Irradiation

In the absence of visible light activity is the major drawback of the pristine TiO_2_, while the zero band-gap structure of graphene makes it an ideal sensitizer. Park et al. revealed the views of generation and collection processes of the photocurrent in a single-layer graphene sample under 514 nm laser light irradiation [117]. Mai et al. calculated the scale of the photocurrent when a clean graphene sample was irradiated by a monochromatic visible light in theory [118]. Although the above results and following reports demonstrate the feasibility of the graphene-TiO_2_ composite photocatalyst working under visible light irradiation, two questions are still unsolved: the electron transport from graphene into TiO_2_ can be achieved by which path? How about the transport probability?

**Figure 4:**
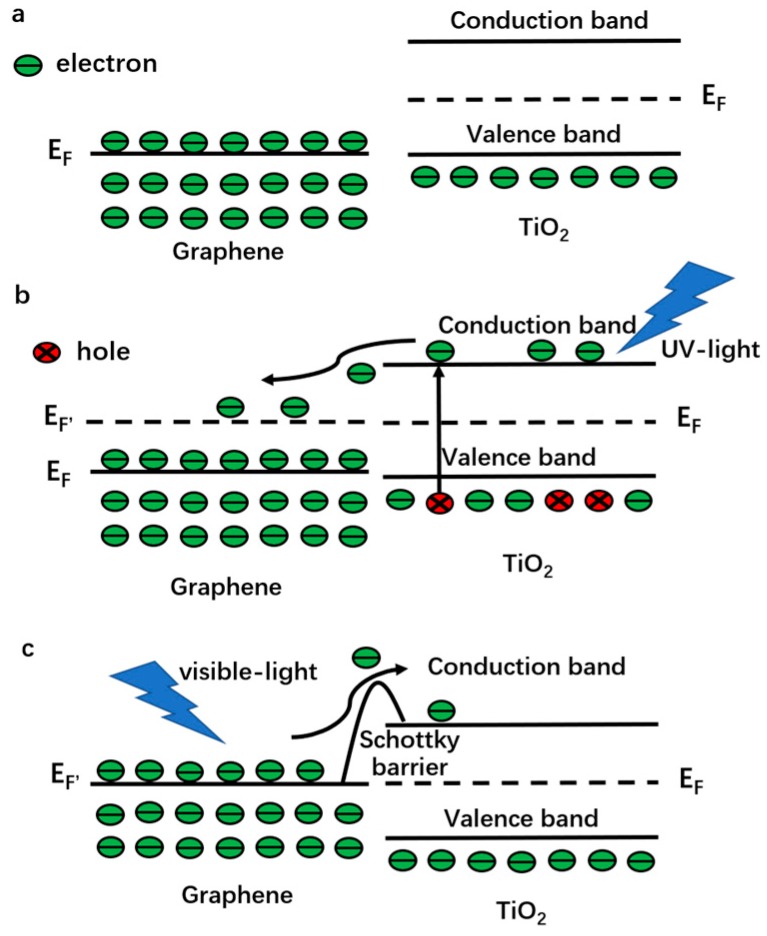
Band structures and interface interactions of the graphene-TiO_2_ (**a**) before combination (**b**) under UV-light irradiation and (**c**) under visible light irradiation with a close chemical contact.

**Figure 5:**
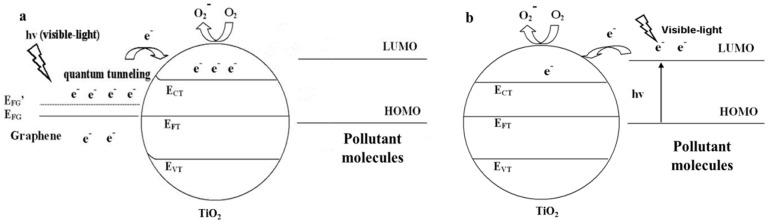
Photocatalytic mechanisms of the (**a**) graphene-TiO_2_ composite and (**b**) self-degradation of dye with pure TiO_2_ under visible light irradiation. Reproduced with permission from [14]. Copyright Elsevier, 2013.

The band structures of graphene and TiO_2_ under various conditions are shown in the Figure 4a–c. As for the pristine materials (before contact), the Fermi levels are 0 V vs. Normal Hydrogen Electrode (NHE) for graphene and 0.5 V vs. NHE for TiO_2_ (Figure 4a). A metal-semiconductor contact (junction) forms at the interface (graphene is a semimetal with a zero band-gap structure) after combining them [14] and the thermal equilibrium would lead to a Fermi level constant throughout the composite. Therefore, electrons will transport into the Fermi level of graphene from conduction band of TiO_2_ spontaneously without illumination or under UV-light irradiation (Figure 4b) [59]. Then, a Schottky barrier and a built-in potential barrier form on the side of graphene and TiO_2_, respectively. Under visible light illumination, a great number of electrons are excited on the Fermi level of graphene and the Schottky barrier must be overcome (quantum tunneling) before these electrons inject into the conduction band of TiO_2_ (Figure 4c). If the metal-semiconductor junction is composited with bulk materials, a wide enough depletion layer appears and hinders the electron transport. However, as for the graphene-TiO_2_ composite, no significant band bending occurs because their sizes are far smaller than that of the space-charge region (the barrier width is limited) [119,120]. Therefore, photo-generated electrons excited in graphene can inject into TiO_2_ by quantum tunneling and thermionic emission to conquer the thin Schottky barrier. At room temperature, the thermionic emission (thermal voltage ~26 meV) is too small to achieve the transfer due to the higher barrier (Schottky barrier height is equal to the difference between the work function of graphene and the electron affinity of TiO_2_, ~2 eV) [121]. Consequently, the quantum tunneling is the sole path to realize the electron transport. Width of Schottky barrier is not greater than the thickness of the adopted graphene (always less than 3 layers, ~1 nm), which is much shorter than the mean free path of electrons in graphene and TiO_2_, indicating no significant additional collision (such as electron-electron or electron-phonon) takes place. Therefore, the major obstruction is Schottky barrier for the electron transport from graphene to TiO_2_ and the probability can be calculated by the following equation:

(1) $$\Gamma =\exp\left\{-\frac{2}{\hbar }\int_{0}^{d}\sqrt{2m(U_{0}-E)}dx\right\}$$

therein, *m* represents electron mass, *E* is kinetic energy of photo-induced electrons, *d* and *U*_0_ are the width and height of the Schottky barrier, ℏ is reduced Planck constant. Before calculating a specific probability, the values of *E* and *U*_0_ should be given. In order to conservatively estimate the probability, two extreme assumptions can be made. Firstly, the energies of all the photo-induced electrons are deemed as *E_F_* (intrinsic Fermi level of graphene), therefore, the Schottky barrier is a constant (~2 eV). In fact, the energies of photo-induced electrons in graphene are higher than *E_F_* because of Pauli exclusion principle (the actual Schottky barrier is small than 2 eV) [14]. Moreover, kinetic energy *E* is considered as zero for all the photo-generated electrons, which also leads to an underestimated transport probability. By adopting these assumptions, the tunneling probability is 5.21 × 10^−7^ when the adopted graphene is ~1 nm in thickness (more than 10^11^ electrons can inject into TiO_2_ from graphene every minute when the intensity of incident light is 100 mW·cm^−2^) [14]. Based on the above discussion, the photocatalytic mechanism of the graphene-TiO_2_ composite photocatalysts is displayed in Figure 5a (the self-degradation mechanism of dye under visible light irradiation with the pure TiO_2_ is shown in Figure 5b for comparison). It is worth noting that a close chemical contact at the interface between graphene and TiO_2_ is the pre-condition for the tunneling behavior. An FTIR curve is a useful tool with which to judge the presence of a strong chemical bond and a new absorption band, resulting from the vibration of Ti-O-C, located at ~800 cm^−1^ can be found [3,54,122].

### 2.3. Characterization Approaches

Besides pollutant decomposition experiments, X-ray diffraction (XRD), Scanning electron microscope (SEM), Atomic force microscope (AFM), X-ray photoelectron spectroscopy (XPS), Fourier transform infrared spectroscopy (FTIR) and Raman spectra, some novel characterization technologies can be used to judge the photocatalytic performance of the graphene modified TiO_2_, including photoluminescence (PL) spectrum (QM4CW, Photon Technology International, Birmingham, NJ, USA), Electron paramagnetic resonance (EPR) spectroscopy (EPR-8, Bruker BioSpin Corp, Rheinstetten, Germany), and Scanning tunneling microscope (STM) (DI Corp, Bakersfield, CA, USA).

**Figure 6:**
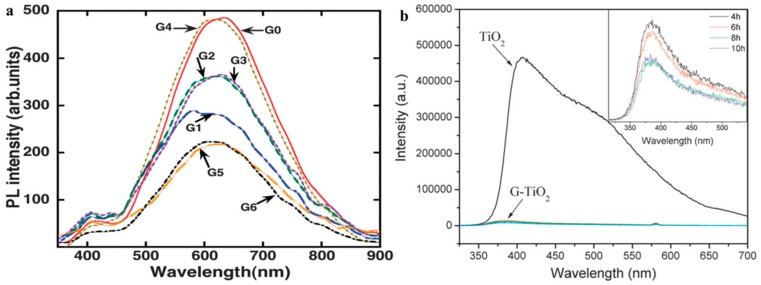
PL spectra of (**a**) pure TiO_2_ and the graphene-TiO_2_ composite, the inset displays corresponding curve of composites by using graphene with various reduction degrees; Reproduced with permission [131]. Copyright RSC, 2013; (**b**) different samples, the G0, G1, G2, G3, G4, G5 and G6 represent bare TiO_2_, transferred graphene-TiO_2_, transfer free graphene-TiO_2_, RGO-TiO_2,_ GO-TiO_2_, graphitic carbon-TiO_2_ and Ti-graphitic carbon-TiO_2_, respectively. Reproduced with permission [132]. Copyright RSC, 2011.

PL spectrum is a highly sensitive tool to study photo-physics of the photo-generated species [123]. In the photocatalysis field, PL curves can be used to analyze the recombination of the electron-hole pairs in the TiO_2_ [124,125,126,127,128]. Melnyk et al. studied the PL spectrum of TiO_2_ with two polydisperse modifications (anatase and rutile) under a low temperature [127]. Sekiya’s group reported time-resolved PL spectra of anatase single crystal samples [128]. Under UV-light irradiation, the origin of the signal peak in visible area from PL profile is attributed to the radiative recombination of the self-trapped excitons in TiO_2_ [129,130]_._ Therefore, PL spectrum is a direct technology to detect the recombination efficiency of the electron-hole pairs. According to the research results from Sellappan group, Zhu’s group and our group, the luminescence efficiency of the graphene-TiO_2_ composite is much lower than that of the bare TiO_2_, indicating the depressed recombination of the electron-hole pairs (Figure 6) [14,131,132]. However, decreased PL intensity not means equivalent increment of strong oxidizing radicals ($O_{2}^{-}$ and ${\mathrm{OH}}^{\cdot }$) because some other possible reasons can bring about non-radiative charge carrier leakage at the interface, such as defect and phonon scattering [131]. Therefore, PL results can be utilized as an indirect evidence to prove the enhanced photocatalytic performance of the graphene-TiO_2_ composite photocatalysts. Moreover, it is worth noting that PL spectra only can be used for the case of UV-light irradiation because of the wide band-gap of TiO_2_.

**Figure 7:**
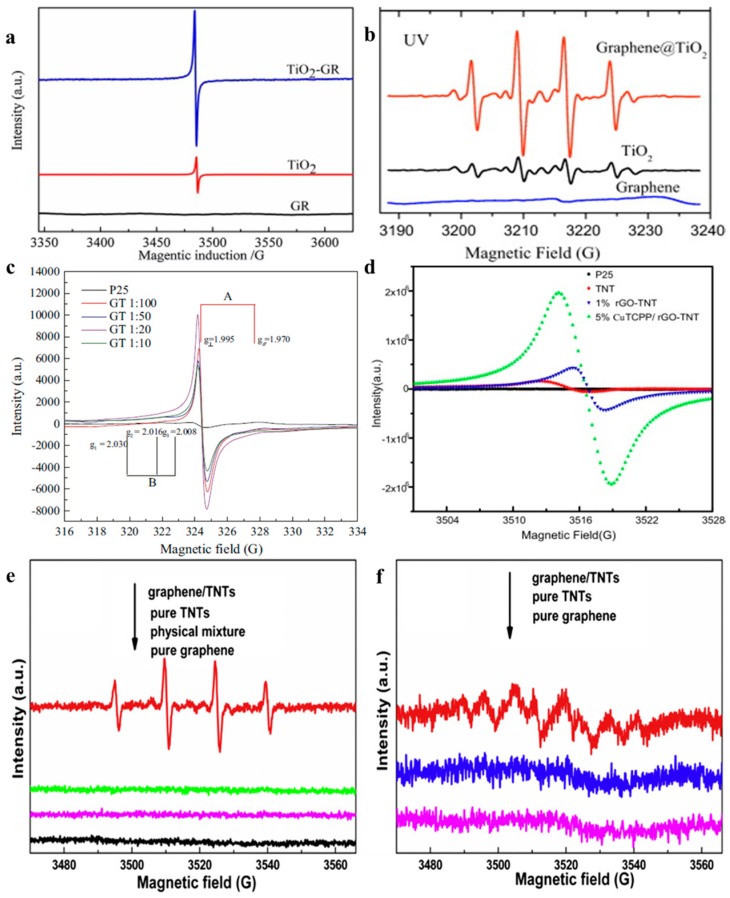
Under UV-light irradiation, the EPR curves (DMPO-${\mathrm{OH}}^{\cdot }$) of TiO_2_ and the graphene-TiO_2_ composite photocatalyst (**a**–**d**), under visible light irradiation, the EPR curves of pure TNTs and graphene-TNTs composite photocatalysts (**e**) DMPO-${\mathrm{OH}}^{\cdot }$ and (**f**) DMPO-$O_{2}^{-}$. Figure 7a Reproduced with permission form [133]. Copyright Elsevier, 2016. Figure 7b Reproduced with permission form [134]. Copyright Elsevier, 2012. Figure 7c Reproduced with permission form [135]. Copyright Elsevier, 2016. Figure 7d Reproduced with permission form [139]. Copyright Elsevier, 2014. Figure 7e,f Reproduced with permission form [14]. Copyright Elsevier, 2013.

EPR spectra can be used to detect the concentrations of the $O_{2}^{-}$ and ${\mathrm{OH}}^{\cdot }$, which are trapped by 5,5-dimethyl-1-pyrroline-*N*-oxide (DMPO) [133,134,135]. The intermittent pulse signal of the DMPO-$O_{2}^{-}$ occurs between 2490–3550 gauss, while continuous wave signal of the DMPO-${\mathrm{OH}}^{\cdot }$ appears in the magnetic field strength range of 3480–3550 gauss. Therefore, the outputs of strong oxidizing radicals can be directly recorded by the EPR curve, which determines the photocatalytic performance of the resulting photocatalyst. Under UV-light irradiation, the signal intensity acts as the criterion to judge the photocatalytic performances of various photocatalysts. In the presence of corresponding signal under visible light illumination can be used to prove the sensitization of graphene, while the signal intensity is closely related to the resulting visible light activity. Therefore, the EPR spectrum is a powerful tool to directly estimate the photocatalytic performances of graphene-TiO_2_ under both UV- and visible light irradiation. The reports from Zhang’s group, Chen’s group, Wan’s group, Dai’s group and our group confirm the above analysis (Figure 7) [14,133,134,135,136,137,138,139]. 

Worth to note that graphene possesses a high stability with these strong oxidizing radicals ($O_{2}^{-}$ and ${\mathrm{OH}}^{\cdot }$) during the photocatalytic reaction, which is confirmed by the high photocatalytic performance after cycle use. In general, the decomposition rate constants of various pollutants maintain more than 90% after repeated use compared with the first performance [3,14,15,55]. Moreover, no obvious change can be seen from the morphology of graphene in the composite photocatalysts after photocatalytic reaction. Wang et al. reported that the photodegradation rate of pollution does not show an obvious decrease during five successive cycles [86]. Xu’s group found that the graphene-TiO_2_ composite possesses a high stability, which is even better than that of the bare TiO_2_ [140]. Our group fabricated graphene-TiO_2_ composite photocatalyst and the light activity maintains ~95% after 20 cycles (phenol, methyl orange and rhodamine are used as the pollution) [3,14,15,55]. All the above reports indicate that the photocatalytic performance of the graphene-TiO_2_ composite is stable, confirming the graphene is stable during the photocatalytic reaction (do not react with $O_{2}^{-}$ and ${\mathrm{OH}}^{\cdot }$) [3,14,15,94,140,141].

**Figure 8:**
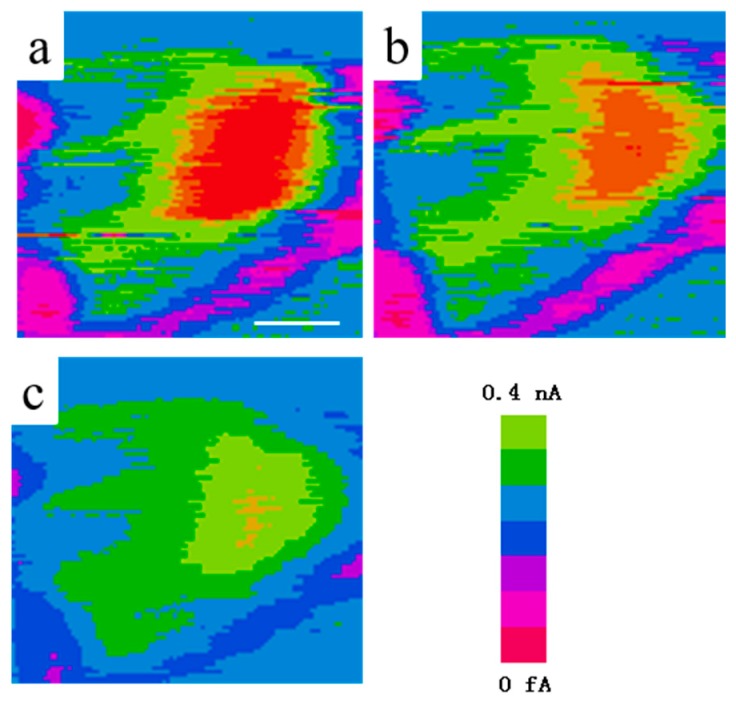
STM images of the graphene-TNTs composite (**a**) without illumination; (**b**) under visible light illumination and (**c**) under UV-light irradiation. The scale bar is 20 nm. Reproduced with permission from [14]. Copyright Elsevier, 2013.

**Figure 9:**
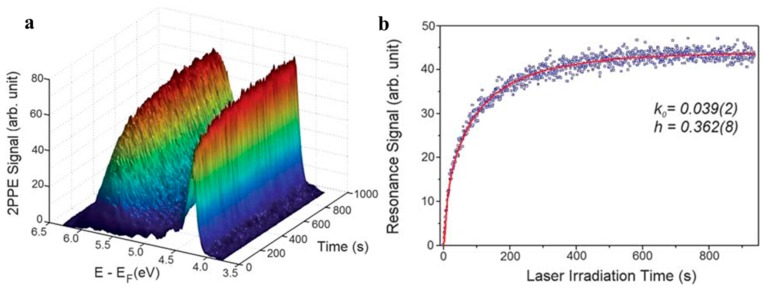
(**a**) Time-dependent two-photon photoemission spectra were measured for the freshly CH_3_OH adsorbed stoichiometric TiO_2_ (110) surface after it had been exposed for different time durations. (**a**) This plot shows the evolution of the time-dependent two-photon photoemission spectra after the surface was exposed for certain time durations; (**b**) The time dependent signal of the excited resonance feature between 4.9 and 6.1 eV measured with the laser power of 64 mW. Reproduced with permission from [138]. Copyright RSC, 2010.

STM was introduced to directly prove the electron transport from graphene to TiO_2_ by our group [14]. As we known, STM is based on the quantum tunnel effect to detect electron states density around the Fermi level of conductive materials [14,136,137]. Therefore, the fluctuation of electron states densities under various conditions (weather with irradiation) in graphene and TiO_2_ can be monitored. Different from SEM image, the STM images are derived from the electron states density around the Fermi level rather than secondary electron from the surface of samples. Therefore, color but not morphology is the basis to identify the graphene and TiO_2_ in the composites, which is the major shortage for this technology. In STM image, lighter color represents higher electron state density and the blue background results from the highly oriented graphite substrate. The direction of electron transport between TiO_2_ and graphene under illumination can be judged by the change of their colors. Our group found that the lighten color of TiO_2_ in a composite photocatalyst under visible light irradiation, proving the electron transport from graphene to TiO_2_ (Figure 8) [14]. Further, Yang’s group designed an ingenious test from the time-dependent two-photon photoemission combining with the STM. STM tip induces molecular manipulation before and after UV-light illumination and the bond cleavage of methanol can be observed (Figure 9) [138]. STM technology provides a direct method to reveal the electron transport direction between the graphene and TiO_2_ under various illumination conditions.

## 3. Optimizing of the Graphene-TiO_2_ Composite Photocatalyst

Although graphene based TiO_2_ composite photocatalysts display numbers of advantages compared with other modifiers in the theory, the reported photocatalytic performances are much lower than predicted values [62,89,90,91,92]. In order to achieve the practical application of this kind of photocatalysts, some attempts have been carried out. During all the optimizing approaches, three categories can be abstracted, which will be discussed as following. 

### 3.1. Mass Fraction of Graphene

**Figure 10:**
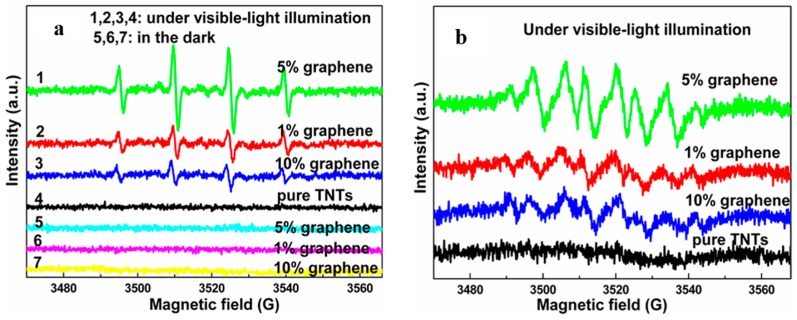
Relationship between mass fraction of graphene and output of the free radicals (**a**) DMPO-${\mathrm{OH}}^{\cdot }$ and (**b**) DMPO-$O_{2}^{-}$. Reproduced with permission from [14]. Copyright, Elsevier, 2013.

Liu et al. Yu et al. and Zhang et al. found that the mass fraction of graphene in the composite photocatalyst is closely related to the resulting photocatalytic performance [139,142,143]. Generally, 1–5 wt % is the recommended proportion of graphene and a synergistic effect is revealed (the EPR is used to judge the photocatalytic performance, Figure 10) [14,15,55]. Under UV-light irradiation, insufficient graphene could not provide an enough big tank to storage the photo-induced electrons transferred from TiO_2_, while excessive black graphene influences the output of the photo-induced electrons in TiO_2_ by absorbing part incident light and producing additional heat. In the case of under visible light illumination, the sensitization is deficient when the content of graphene is too low. Contrarily, increased mass fraction of graphene could not continuously improve the visible light activity of the resulting composite photocatalysts because only the electrons injected into the conduction band of TiO_2_ can produce corresponding strong oxidizing radicals. Therefore, achieving the synergy between the mass fraction and positive effects of graphene is significant to the resulting high performances. Moreover, it is easy to understand the diversity of the recommended mass fraction values of graphene from various groups by considering the distinction of morphology, thickness and quality of the adopted RGO samples for the resulting composite photocatalysts.

### 3.2. Morphologies of TiO_2_ and Graphene

**Figure 11:**
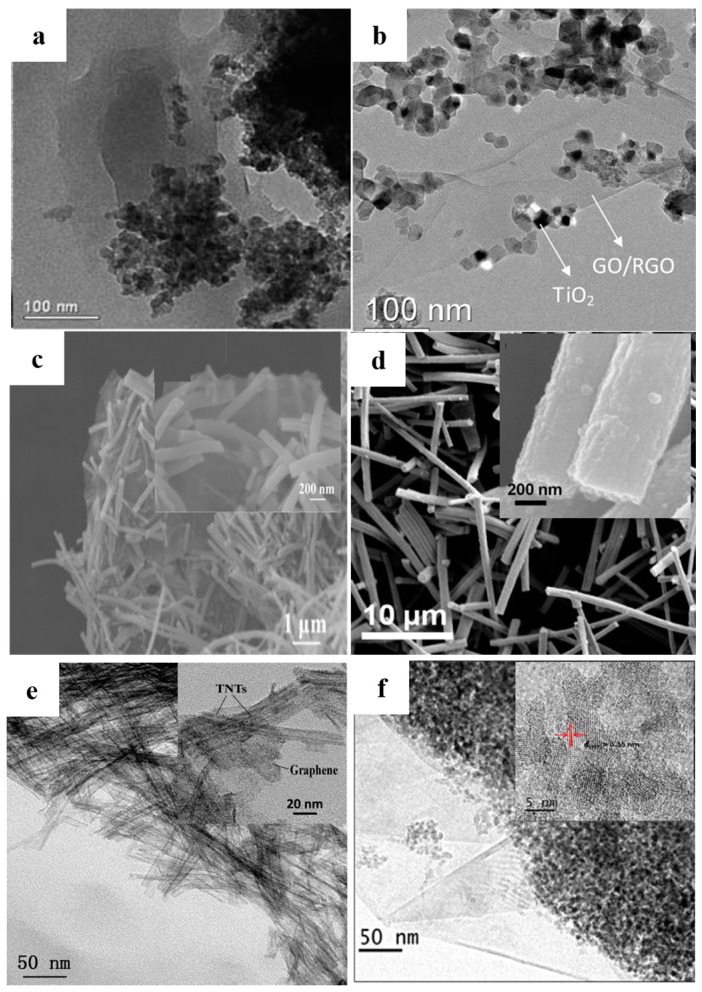
Graphene-TiO_2_ composite photocatalysts with various morphologies of TiO_2_, (**a**,**b**) TiO_2_ nanoparticles Reproduced with permission [83] Copyright RSC 2010. (**c**) TiO_2_ nanotubes Reproduced with permission [105] Copyright Elsevier 2014. (**d**) TiO_2_ nanofibers Reproduced with permission [106] Copyright Elsevier 2014 (**e**) TNTs Reproduced with permission [15] Copyright Elsevier 2011 (**f**) TiO_2_@graphene, insets are the high magnification images. Reproduced with permission [108] Copyright Elsevier 2014

BET area is regarded as an important parameter to determine the adsorption ability of photocatalysts. Because of the high surface area to body weight ratio of nano materials, sizes of raw materials (including TiO_2_ and graphene) are always limited to tens of nanometers. However, two shortages greatly restrict the high BET area of the resulting composite photocatalysts. Firstly, the serious stacking behavior of 2D RGO nanosheets leading to the practical BET area is only one-fiftieth of the theoretical value (only the surface graphene makes contribution to the adsorption ability). Moreover, the discrete TiO_2_ nanoparticles tend to an agglomerate behavior during the hydrothermal reaction, which also exerts a negative effect on the resulting adsorbability. Generally, the reported BET areas of the graphene-TiO_2_ composite photocatalysts are always less than 50 m^2^·g^−1^ (Figure 11a,b) (without any optimizing) [83,85]. 

**Figure 12:**
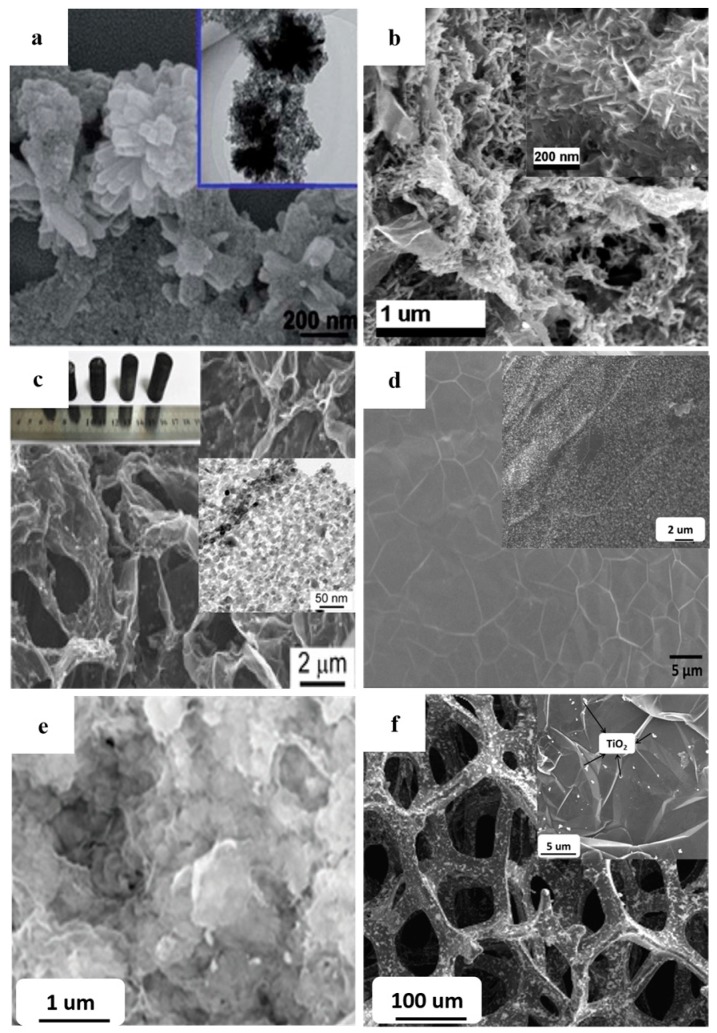
The 3D graphene-TiO_2_ composite photocatalysts with various morphologies of graphene, insets are the high magnification images. (**a**–**e**) 3D graphene aerogel prepared by RGO (**f**) 3D graphene network prepared by CVD method. Figure 12a,b Reproduced with permission [8] Copyright Elsevier, 2017. Figure 12c Reproduced with permission [110] Copyright ACS, 2016. Figure 12d Reproduced with permission [111] Copyright ACS, 2013. Figure 12e Reproduced with permission [112] Copyright ACS, 2016. Figure 12f Reproduced with permission [55] Copyright Elsevier, 2017.

In order to avoid the excessive agglomeration, TiO_2_ (including TiO_2_-like materials) with various shapes were designed. By using the –COOH and –NH_2_ functionalized RGO nanosheets as the shape controller, Sordello et al. prepared the high BET area RGO-TiO_2_ composite photocatalysts with a controllable morphology and crystal facets [144]. Perera et al. Li et al. and Kim et al. prepared the RGO-TiO_2_ nanotube/nanofiber composites by the hydrothermal method and the large BET area brings about a high photocatalytic performance (Figure 11c,d) [104,105,106]. Our group prepared the RGO-TNTs composite photocatalyst and the tubular construction of the TNTs endows a large BET area for the resulting photocatalyst (~300 m^2^·g^−1^). The decomposition rate constant of rhodamine-B (RB) is much higher than (~5 times) that of the reported graphene-TiO_2_ nanoparticles photocatalysts (the optimized mass fraction of the RGO is 5 wt %) (Figure 11e) [15]. Moreover, the core-shell constructed graphene-TiO_2_ composites also display a high photocatalytic performance due to its increased BET area (Figure 11f) [108]. Besides morphology of TiO_2_, corresponding optimization of graphene also implements a remarkable influence on the resulting BET area by depressing the stacking behavior. Wang et al. adopted the CNTs acting as the marble pillar to construct a 3D structure with the RGO nanosheets and TiO_2_, the degradation rate of MB increases 2.2 times compared with that case of adopting an unmodified photocatalyst [145]. Although this 3D structure efficiently inhibits the excessive stacking of the RGO nanosheets, the direction of the CNTs is difficult to control. Zhang et al. adopted a one-pot route to achieve the formation and combination of the 3DRGO aerogel and TiO_2_ and the resulting high pore volume and large BET area bring about a high adsorption capacity and the similar composites have been reported by Yan et al. Ding et al. Zhang et al. and Park et al. (Figure 12a–e) [8,110,111,112,146]. Moreover, Zhong et al. further utilized the 3DRGO-TiO_2_ aerogel as a carrier to load MoS_2_ nanosheets for a co-catalyst, achieving a significant enhancement in adsorbability [147]. Although the 3DRGO-TiO_2_ photocatalyst displays a large BET area and an improved adsorbability, the uncontrollable thickness (which is closely related to the BET area of graphene) and a high defect density (decrease the lifetime of electrons) of the 3DRGO hinder the further improvement of the resulting photocatalytic performances. Contrarily, CVD is a relatively convenient method to fabricate the high-quality 3DGNs with a controlled thickness and a large BET area. Recently, our group prepared the thickness controllable 3DGNs by adjusting the CH_4_ and H_2_ flows during the CVD process and the BET area is as high as ~500 m^2^·g^−1^ for the resulting 3DGNs-TiO_2_ composite photocatalyst (Figure 12f) [53,55,148]. The decomposition rate constant of phenol is ~5 times higher than the previous reported results and the bi-layer constructed 3DGNs is found the best choice resulting from the following reason. The integrity and continuity of a monolayer 3DGNs sample is difficult to satisfy, while thicker sample could not provide more assistance for the adsorption function (only surface graphene is contributed). In fact, the 3D structure of graphene not only enhances the adsorption ability of the photocatalysts but also in favor of the uniform distribution of TiO_2_ nanoparticles (increasing the contact area between them). Therefore, the 3DGNs shows a promising potential in the photocatalysis field and the following reports from Yan’s group and Yu’s group confirmed the conclusion [149,150]. Moreover, Cui’s group fabricated the graphene-TiO_2_ multilayer films by CVD and magnetron sputtering methods, which further improves the contact area between the graphene basal plane and TiO_2_ [151]. The sufficient contact between the graphene and TiO_2_ not only provides more opportunities for electron transport but also achieves the synergy of these two materials.

### 3.3. Quality of Graphene

The high photocatalytic performance of photocatalyst is closely related to the utilization of photo-induced electrons and two factors including electron transport and electron-hole pairs recombination determine the resulting utilization efficiency of photo-generated electrons. As for the case of UV-light irradiation, how to depress the recombination of electron-hole pairs is the major optimization approach. For the case of visible light irradiation, besides suppressing the recombination behavior, how to enhance the electron transport at the interface between graphene and TiO_2_ is quite important due to the presence of a Schottky barrier. Therefore, decreasing the width of this barrier is vital for their visible light activities. Various defects of TiO_2_ and graphene always act as recombination centers to the electron-hole pairs. Therefore, the quality of them is closely related to the resulting photocatalytic performances. Because commercial TiO_2_ is used as the raw material in most reports, only the influences from the quality evolution of graphene is discussed here. 

Two types of defects can be classified in the RGO, including structure defects and additional functional groups. The former is introduced during the violent redox and exfoliation procedure, such as edge, carbon vacancy and pentagon (heptagon) structure. The latter, including hydroxyl, carboxyl and epoxide groups, is resulted from the strong oxidant to intercalate laminar graphite structure and the total amount is controllable by adjusting the reduction time [54]. Both the two types of defects shorten electron lifetime by destroying the nonlocal *π* electron orbit of graphene, which adverse to the high yield of the strong oxidizing radicals [152,153]. Therefore, the high quality of graphene is rather important for the resulting performance of the composite photocatalysts. Xu’s group proposed a solvent exfoliated method to prepare the high quality RGO to combine with TiO_2_ and the resulting photocatalytic performance enhances because of the prolonged electron lifetime [154]. Similarly, Gray’s group reported that the photocatalytic reduction of CO_2_ significantly enhances when minimizing the RGO defects is achieved [155]. With the continuous research, surface functional groups of the RGO are found playing a positive role, simultaneously. These surface functional groups act as a bridge to link the graphene basal plane and TiO_2_ (chemical contact), achieving the *π*-*d* electron coupling and the following electron transport [50,54]. Therefore, different from the structure defects, a moderate amount of surface functional groups of the RGO is helpful. Elimelech’s group reviewed the influences from functional groups of the RGO on the resulting photocatalytic performances and the corresponding positive effect is emphasized [156]. Insufficient surface functional group could not provide enough channels for the electron transport, while excessive functional groups decrease the intrinsic electrical property of graphene (shorten the electron lifetime). Moreover, our group found that a moderate surface functional group amount of the RGO is beneficial to the chemical contact between graphene and epoxy resin, indicating the functional groups can be utilized to achieve the infiltration of graphene in other materials (including organics and inorganics) [54]. 

As for the case of the 3DGNs based photocatalysts, in the absence of functional group on its surface (because of the CVD preparation method) means the chemical contact between the graphene basal plane and TiO_2_ must be achieved by other ways. Our group found that the surface defects of the 3DGNs play as the bridge and the 3DGNs with moderate defects assisted composite photocatalysts displays the highest performances. On one hand, surface defects of the 3DGNs exert a negative influence to shorten the electron lifetime (reduce the yield of the $O_{2}^{-}$ and ${\mathrm{OH}}^{\cdot }$). On the other hand, these defects impose a positive effect to achieve the *π*-*d* electron coupling (enhancing the electron transport between the graphene basal plane and TiO_2_), simultaneously. Therefore, the 3DGNs with a moderate defect density can realize the balance and endow the best performance for the resulting photocatalysts. Because only the surface defect is useful, the thickness of the 3DGNs becomes another key parameter. Sample with a bi-layer thickness is recommended because all the defects are on the surface (a thinner 3DGNs sample possesses a larger BET area, however, the integrity of the monolayer sample is unsatisfied, which leads to other negative effects).

## 4. Prospective

Direct determinants of photocatalytic performances of the graphene-TiO_2_ composite photocatalysts include the outputs of the strong oxidizing radicals and the adsorption amount of pollutant molecules. Further, the outputs of the $O_{2}^{-}$ and ${\mathrm{OH}}^{\cdot }$ are closely related to the lifetime of the photo-induced electrons, which is dependent on the recombination rate of the electron-hole pairs and the mean free path of electrons in graphene and TiO_2_. Moreover, the utilization rate of the photo-induced electrons is significantly affected by the electron transport at the interface between these two materials. Therefore, how to give full play to the functions of graphene (an electron tank under UV-light irradiation and a sensitizer under visible light irradiation) is the pivotal issue. As for the adsorption ability, although BET area of a photocatalyst exerts a significant influence, the efficiently adsorbability (chemisorption) is the determinant. In fact, optimizing the surface chemistry of the adopted graphene (the defect density of the 3DGNs and the residual amount of surface functional groups of the RGO nanosheets) is the fundamental approach to enhance the chemisorption ability. The specific relationship between the photocatalytic performance and influence factors (including improvement methods) are shown in Table 1.

### 4.1. How to Enhance the Chemisorption Ability of the Graphene-TiO_2_ Photocatalysts?

**Figure 13:**
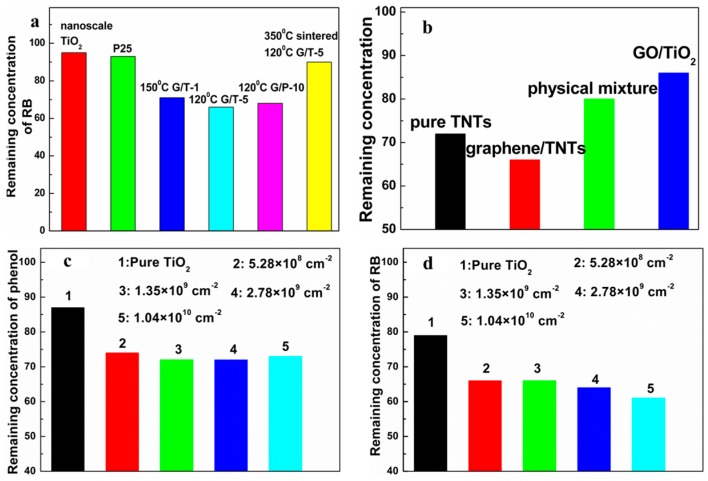
Relationship between adsorption ability and (**a**) mass fraction of graphene (graphene-TNTs composites), Reproduced with permission [15]. Copyright 2011 Elsevier (**b**) various composites (graphene-TNTs, graphene oxide-TNTs and physical mixture), Reproduced with permission [15]. Copyright 2011 Elsevier (**c**,**d**) defect density of 3DGNs (3DGNs-TiO_2_ composites). Reproduced with permission [3]. Copyright 2017 Elsevier The model pollutant is rhodamine-B in (**a**,**d**) and phenol (**b**,**c**).

Adsorption experiments manifest that BET area determines the adsorption ability of a photocatalyst [8,63,64,142,143,144,146]. However, a large BET area not equals a good photocatalytic performance because only the chemisorbed pollutants can be decomposed [157,158,159]. A series of the 3DGNs-TiO_2_ composite photocatalysts by adopting the 3DGNs (bi-layer thickness) with various defect densities were prepared by our group and all samples possess similar BET area and total adsorption ability at room temperature (Figure 13, including physical adsorption and chemical adsorption, phenol and methyl orange were used as model pollutants, an agitating process with 20 min in the dark was performed to achieve the adsorption balance). In order to abstract the chemisorption ability, more adsorption experiments were carried out at high temperature. Under high temperature (>80 °C), chemical adsorbed pollutants retain on the surface, while physical adsorbed molecules fall off due to their enhanced kinetic energy. Various residual amounts of pollutants in the solutions by using these composite photocatalysts under high temperature indicate the defect density of the 3DGNs imposes a remarkable effect to the resulting chemisorption ability. Similarly, the surface functional group of the RGO should exert a significant influence on the chemisorption ability of the composite photocatalysts. Actually, the chemical adsorption can be deemed to form a chemical bond between the photocatalyst and pollutant molecules. Because the interaction between the sp^2^ bonded carbon atoms (graphene basal plane) and pollutant molecules is weak (*π*-*π* conjugation or Van der Waals force), additional functional groups (or defects) of graphene enhance the chemisorption. However, the corresponding research on revealing the relationship between the functional group amount (and types) of the RGO and the resulting chemisorption ability of the RGO-TiO_2_ composite photocatalyst is insufficient.

In the future, the major attention should be focused on how to promote the chemical contact between graphene and pollutants. As for the RGO-TiO_2_ composites, the bonding capability between the surface functional group (−OH, −OOH, =O) and various pollutants molecules deserves to reveal, which is valuable to design the proper RGO-TiO_2_ photocatalyst for specific pollutants. As for the 3DGNs-TiO_2_ composites, the defect density of the 3DGNs deserves further optimizing. Moreover, the theoretical calculation on pollutants adsorption of the graphene-TiO_2_ is insufficient, lacking enough analog data to support the experimental results. On the other hand, although the major contribution of the adsorption ability results from graphene, corresponding optimizing on depressing the agglomeration of TiO_2_ also exert a positive effect to adsorb more pollutants. Cai’s group and our group found that TiO_2_ with a low loading amount is beneficial for depressing the agglomeration behavior [149,160]. Moreover, searching proper dispersing agents is one of feasible approaches for the further study.

### 4.2. How to Depress the Recombination of the Electron-Hole Pairs in TiO_2_ under UV-Light Irradiation?

As discussed in Section 2.2.1, one of major functions of graphene in the composite photocatalyst under UV-light irradiation is that this material acts as an electron tank to accept the photo-induced electrons transported from TiO_2_ [161,162]. The storage amount and transfer velocity determine the performances of graphene and the resulting photocatalysts. In other words, the suppression of the recombination of electron-hole pairs is the key factor for determining the resulting photocatalytic performance of the photocatalyst under UV-light irradiation.

**Figure 14:**
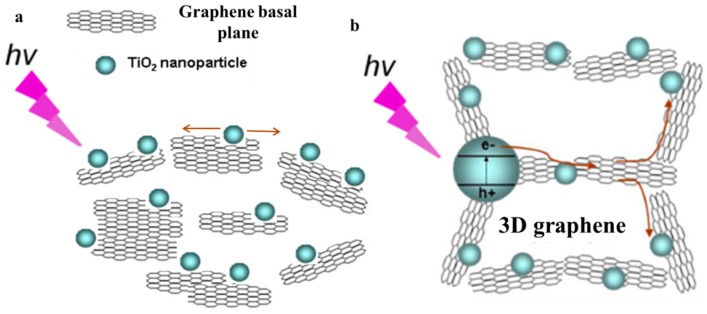
Schematic diagram of electron transport in the (**a**) 2D graphene-TiO_2_ composite and (**b**) 3D graphene-TiO_2_ composite. Reproduced with permission from [111]. Copyright ACS, 2013.

Kongkanand et al. reported that ~32 carbon atoms in the perfect CNTs and graphene can accept one foreign electron [163]. Therefore, the storage ability of the photo-induced electrons is decided by the mass fraction of graphene in the composite [163,164] and the corresponding optimizations have been intensively reported [139,142,143]. However, the obvious difference of recommended graphene content from various groups manifests the morphology, thickness and quality of the adopted graphene also impose a significant influence. In the future, these factors should be taken into account to build a rounded criterion to guide the optimization of graphene mass fraction. Besides electron storage amount, the electron transport velocity also exerts a remarkable influence on the resulting photocatalytic performance, which is determined by the amount of the electron transport channels between graphene and TiO_2_. Therefore, optimizing the morphology of graphene and TiO_2_ to increase the contact area between them is one of development directions. The 3D continuous structure of the 3DGNs (and the 3DRGO aerogel) endows an innate advantage for fast electron transport (Figure 14), the further optimizing for more rational morphologies is necessary. Moreover, developing new additives to achieve the uniform distribution of TiO_2_ nanoparticles on the graphene surface deserve continuous study. In fact, large contact area is the precondition, while the chemical bonding between the graphene basal plane and TiO_2_ is the core factor. As for the cases of the RGO modified samples, further optimizing the amount and type of the surface functional groups is vital to promote the electron transport velocity and output of the $O_{2}^{-}$ and ${\mathrm{OH}}^{\cdot }$. As for the 3DGNs assisted samples, the surface defect density of the 3DGNs is crucial for the electron transport from TiO_2_ to the graphene basal plane. Therefore, providing a proper amount of bridges at the interface between graphene and TiO_2_ is one of the objectives in the future (excess functional group and surface defect would shorten the electron lifetime due to the degraded electrical property of graphene). Moreover, searching proper linkers to achieve a better chemical contact between them is another feasible method.

### 4.3. How to Promote Electron Transport at the Interface of the Graphene Basal Plane and TiO_2_ under Visible Light Irradiation?

Preparing photocatalysts with a visible light activity is the development tendency because of their wide application range. A Schottky barrier is installed because of the electronic structures of graphene and TiO_2_, which hinders the electron transport from the Fermi level of graphene to conduction band of TiO_2_ under visible light illumination. Therefore, how to diminish the impact from this barrier is the crucial factor to achieve the outstanding sensitization of graphene. Besides the similar optimizing parameters discussed in Section 4.2, the thickness of graphene imposes a significant influence to determine the visible light activity of the resulting composite photocatalyst. Both the promotion of electron transport at the interface and suppression of recombination of electron-hole pairs exert significant influences on the resulting photocatalytic performances under visible light illumination. In fact, these two factors not only determine the photocatalytic properties of graphene-TiO_2_ nanocomposite but also dominate their performance in other solar energy conversion devices [4].

**Figure 15:**
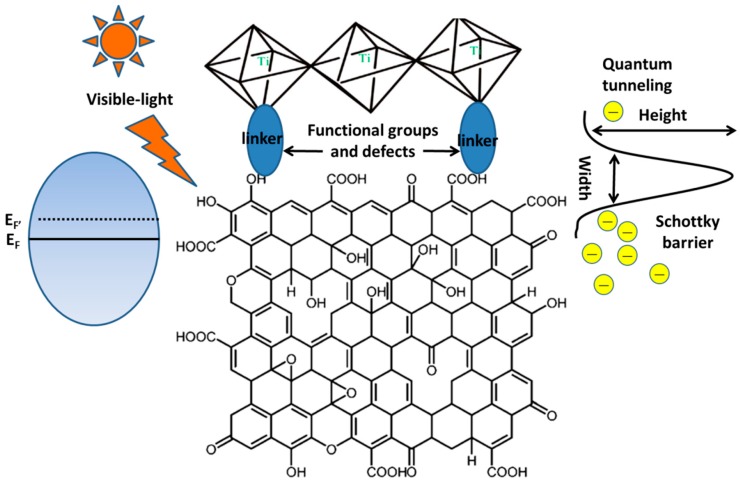
Electron transport between graphene and TiO_2_ at their interface.

According to the corresponding calculation, the tunneling probability of the photo-induced electrons is determined by the height and width of the Schottky barrier [14]. Because of the settled electronic structure of TiO_2_ and graphene, the Schottky barrier height is mixed. Therefore, decrease its width is a reasonable approach to enhance the electron tunneling at their interface (Figure 15). The maximum width of Schottky barrier can be deemed as the thickness of the adopted graphene. Considering the uniform thickness of the RGO nanosheets is difficult to obtain to date, how to prepare the thickness controllable RGO samples and keep their uniform thickness in the following hydrothermal reaction deserve further study. On the other hand, although the thickness of the 3DGNs can be adjusted during the CVD process, the additional control of the surface defect density is needed to ensure the chemical contact between the graphene basal plane and TiO_2_ [55,165,166]. Therefore, a balance between the high electron tunneling probability at the interface and the good intrinsic electrical property of graphene should be achieved by an elaborate design of the 3DGNs defect density in the future (or functional group amount of the RGO). 

Researching the proper dispersing agents to keep the designed thickness of the RGO is one of aims in the future and utilizing electrostatic repulsive force may be a feasible way of doing this. The previous reports indicate that a bi-layer thickness of the 3DGNs is an optimizing structure to electron tunneling and pollutant adsorption. Therefore, how to enhance the output of the 3DGNs with a bi-layer construction also needs the further study. 

## 5. Conclusions

In this progress, some important evolvements of the graphene modified TiO_2_ composite photocatalysts have been presented. Their photocatalytic mechanisms under UV- and visible light irradiation are discussed according to their electronic structures. The large BET area (providing more active adsorption sites), zero band-gap (acting as a sensitizer), high electron mobility (prolong electron lifetime) and excellent electron storage ability (playing as an electron tank) in theory of graphene endow it a wonderful modifier for traditional photocatalysts. In order to further enhance the resulting photocatalytic performances of the graphene-TiO_2_ photocatalysts, corresponding optimizations on the mass fraction, morphologies and quality of graphene and TiO_2_ have been carried out, which are discussed and analyzed in this progress. Moreover, a prospective aiming at three core problems of the graphene-TiO_2_ composite photocatalysts is provided. Specially, the corresponding discussion of the 3DGNs assisted samples is emphasized. Moreover, some novel technologies on estimating the resulting photocatalytic performances are also discussed here, including the STM, EPR and PL spectra. Although some bottlenecks of the graphene-TiO_2_ composite photocatalyst still occur, the promising prospects inspire researches continuous modifications and innovations.

## Figures and Tables

**Table 1:** Relationship between photocatalytic performances and optimization technologies.

Bottlenecks of Pure TiO_2_	Solution	Essential Reason	Specific Optimizing	Relationship
Lacking visible light activity	Sensitization of graphene	Exciting photo-induced electron under visible light irradiation	Optimizing thickness of graphene	Thinner graphene brings about a higher quantum tunneling probability
			Optimizing functional group amount and types of the RGO	A higher defect density or functional group amount is beneficial to a higher tunneling probability
	Providing electron transport channels from graphene to TiO_2_	Achieving the transfer of photo-induced electrons	Optimizing defect density of the 3DGNs	
High electron-hole recombination rate	Improving quality of graphene	Enhancing the mean free path of electron	Optimizing the preparation process of graphene and resulting composite photocatalysts	A higher quality brings about a higher mean free path
	Achieving the fast electron transport between graphene and TiO_2_	Prolong the lifetime of electrons	Optimizing the morphology and defect density (or functional group amount) of graphene	A higher defect density or functional group amount provides more transport channels
Low chemical adsorption ability	Increasing the BET area	Providing more adsorption sites	Optimizing the morphology of graphene and TiO_2_	Graphene and TiO_2_ with 3D construction is beneficial to a higher BET area
	Increasing chemisorption active sites	Promote the formation of chemical bond between graphene and TiO_2_	Optimizing defect density (or functional group amount and type) of graphene modifier	A higher defect density or functional group amount provides more chemisorption active sites
